# Sequential effects in continued visual search: Using fixation-related potentials to compare distractor processing before and after target detection

**DOI:** 10.1111/psyp.12062

**Published:** 2014-02-11

**Authors:** Christof Körner, Verena Braunstein, Matthias Stangl, Alois Schlögl, Christa Neuper, Anja Ischebeck

**Affiliations:** a Department of Psychology, University of GrazGraz, Austria; b Institute of Science and Technology KlosterneuburgKlosterneuburg, Austria; c Institute for Human-Computer Interfaces, University of Technology GrazGraz, Austria; d Institute for Knowledge Discovery, University of Technology GrazGraz, Austria

**Keywords:** Serial visual search, ERP, Eye movements, Fixation-related potential, Sequential effects

## Abstract

To search for a target in a complex environment is an everyday behavior that ends with finding the target. When we search for two identical targets, however, we must continue the search after finding the first target and memorize its location. We used fixation-related potentials to investigate the neural correlates of different stages of the search, that is, before and after finding the first target. Having found the first target influenced subsequent distractor processing. Compared to distractor fixations before the first target fixation, a negative shift was observed for three subsequent distractor fixations. These results suggest that processing a target in continued search modulates the brain's response, either transiently by reflecting temporary working memory processes or permanently by reflecting working memory retention.

Visual search is a behavior that occurs many times every day. When we search for our keys before leaving the house or when we look for a familiar face in a crowd, we perform a visual search, that is, the search for a target object among a set of more or less similar objects or distractors. In a complex visual environment, we are typically not able to spot the target immediately, and have to inspect and classify individual objects one by one—a process known as serial visual search (Treisman & Gelade, 1980). In the present study, we used EEG measurements to investigate how targets and distractors are processed during serial visual search by comparing fixation-related potentials.

A standard serial visual search task consists of displays with targets (target-present) or without targets (target-absent), which are presented to participants in a random order. The participant is asked to press one of two buttons for either target-absent or target-present displays. In target-present displays, the search process can stop as soon as the target is found (self-terminating search). Responses take longer on average in target-absent displays because all items in the display have to be inspected (exhaustive search). This prototypical search paradigm has attracted a great deal of research effort over the past 40 years (see, for a review, Wolfe, 2003).

In the present study, a variation of this paradigm was used. Here, displays with either one or two identical targets were presented, and participants were instructed to manually indicate whether there were one or two targets in the display (Horowitz & Wolfe, 2001; Ward & McClelland, 1989). Consequently, a one-target response can only be given after all items have been inspected. This takes longer on average than searching two-target displays, where a response can be produced as soon as the second target is detected (Gibson, Li, Skow, Brown, & Cooke, 2000; Körner & Gilchrist, 2008). The key difference to the standard search paradigm is that the search has to continue after the first target has been found, effectively decoupling the manual response from the detection of the first target. The multiple-target search paradigm is therefore suited to investigate brain activation for targets and distractors at different stages of the search process. Specifically, it can be investigated how the processing of the first target influences the subsequent processing of distractors.

Because of the high temporal resolution of electroencephalogram (EEG), fixation-related potentials (FRPs) can be used to track changes in processing during serial visual search in real time. To calculate FRPs, we recorded eye movements and locked the EEG signals to the onset of every fixation that occurred within a trial (Kazai & Yagi, 1999; Yagi, 1979). Although the number of studies that have recorded FRPs is limited, FRPs have been applied successfully to word recognition and reading (e.g., Baccino & Manunta, 2005; Dimigen, Sommer, Hohlfeld, Jacobs, & Kliegl, 2011; Hutzler et al., 2007; Simola, Holmqvist, & Lindgren, 2009), pattern reversal (e.g., Kazai & Yagi, 2003), object identification (e.g., Rämä & Baccino, 2010), change detection (Nikolaev, Nakatani, Plomp, Jurica, & van Leeuwen, 2011), and free viewing (e.g., Graupner, Pannasch, & Velichkovsky, 2011). To our knowledge, our experiment is the first to apply FRPs to a serial visual search task.

Brain activity during visual search has been investigated with EEG, typically by averaging brain activity locked to the onset of the display using search displays that can be scanned quickly and without eye movements. In a seminal paper by Luck and Hillyard (1990), the authors proposed that the detection of a target in a serial search task bears a resemblance to the detection of a rare deviant stimulus that occurs unpredictably in a sequence of standard stimuli (“oddball” task). In both tasks, the target (or the deviant) is a stimulus event that occurs rarely compared to distractors (or standard stimuli). The event-related potential (ERP) component that is typically observed in oddball tasks on deviants is a positive-going wave approximately 300 ms after stimulus presentation, referred to as the P3 or P300 (Sutton, Braren, Zubin, & John, 1965). Its amplitude was observed to increase when the probability of a target was decreased (Duncan-Johnson & Donchin, 1977). Luck and Hillyard hypothesized on the basis of these findings that adding distractors to a display in a serial search task would render the early detection of a target more unlikely. They found that the amplitude of the P3 component in target-present trials increased when more distractors were added to the display. On trials with a target-absent display, the smallest amplitude of the P3 component was observed. Although visual search was investigated using EEG in later studies, the bulk of this research has focused on another ERP component, namely, the N2 posterior contralateral (N2pc) component, a negative-going wave observed 200–300 ms after display onset. This component was found to be sensitive to the orienting of covert visual attention to the right or left visual field (Luck & Hillyard, 1994; Woodman & Luck, 1999, 2003).

Most research so far has concentrated on measuring covert attention using displays that can be inspected without eye movements, by calculating ERPs for the first 500 ms from display onset. In the present experiment, however, we set out to explore overt shifts of attention during continued visual search. We allowed participants to move their eyes freely from item to item, recorded their eye movements, and calculated ERPs from the onset of a fixation. Eye movements were recorded to determine whether a distractor or a target was fixated. Care was taken to design a display that ensured the identification of only one item at a time. In particular, we were interested in how the processing of a target differs from the processing of a distractor and how memorizing the detection and location of the first target alters the processing of subsequently fixated distractors.

Having participants move their eyes poses a challenge for ERP analysis because of their influence on the EEG signal (see Dimigen et al., 2011, for a state-of-the-art review). As eye movements are deliberately included in our experiment, we relied on offline correction methods and considered a regression-based correction as well as a correction based on the assumption of independent components. The regression-based correction we used is comparatively mild in its effect on the data and has the advantage of being observer-independent when compared to other methods such as corrections based on the visual inspection of components. Nevertheless, ERPs observed in a fixation-related analysis of EEG data might still contain eye movement residuals when compared to ERP components observed without eye movements. Wherever possible, we therefore analyzed ERPs only during intervals when no eye movements occurred, that is, during periods of fixation, and we are very careful when interpreting our results.

In the multiple-target search task, the search continues after the detection of the first target for several seconds. To produce a correct response, the fact of having found the first target and its location has to be remembered for the remainder of the search. EEG studies investigating visual working memory (VWM) found that the maintenance of information in working memory is associated with a slow negative shift over the length of the retention interval (Barret & Rugg, 1988, 1989). This negative slow wave (NSW) was observed to be sensitive to the type of information (location information vs. object information) retained (Bosch, Mecklinger, & Friederici, 2001; Klaver, Smid, & Heinze, 1999; Ruchkin, Johnson, Grafman, Canoune, & Ritter, 1997), to the amount of information or memory load (e.g., Mecklinger & Pfeifer, 1996), and to the quality of storage (Rösler, Heil, & Röder, 1997). Because our task requires the retention of the position of the first target, we would expect a negative shift on distractor fixations after the first target was fixated.

Finding such a negative shift, or NSW, could also contribute to the controversy about the role of memory in visual search. Horowitz and Wolfe (1998, 2001) have argued that visited distractor locations are not memorized at all in visual search. Recent evidence using behavioral measurements such as eye movements, however, supports the view that at least some distractor positions are memorized to prevent revisiting previously fixated items (e.g., Gilchrist & Harvey, 2000; Körner & Gilchrist, 2007). Electrophysiological correlates associated with retention in visual working memory after having visited the first target would strengthen the view that memory processes play a significant role in visual search.

In the present experiment, we used FRPs to compare the processes related to the fixation of the first target with the processing of the distractor visited immediately before. We also compared FRPs on distractors visited before and after the first target fixation. First, if fixating a target is processed as a rare event, a positive-going wave with a latency in the range of the P3 is expected for target fixations compared to fixations on the last distractor before the first target. Second, if finding the first target triggers retention processes in visual working memory, a slow negative shift should be observed. To perform the task correctly, the location of the first target must be stored in VWM until a response can be produced. We therefore expect a NSW for fixations on distractors that follow the fixation of the first target, when compared to distractor fixations that immediately precede the fixation of the first target.

## Method

### Participants

Twenty-two healthy adults took part in this study. All participants had normal or corrected-to-normal vision. They received monetary compensation for their participation and g**a**ve written informed consent. Due to technical malfunction, the data from three participants could not be analyzed. Two participants had to be excluded from further analysis due to excessive muscular and/or ocular artifacts. The remaining sample consisted of 17 participants (mean age 28.0 years, *SD* = 4.9).

### Stimuli and Procedure

**Figure 1:**
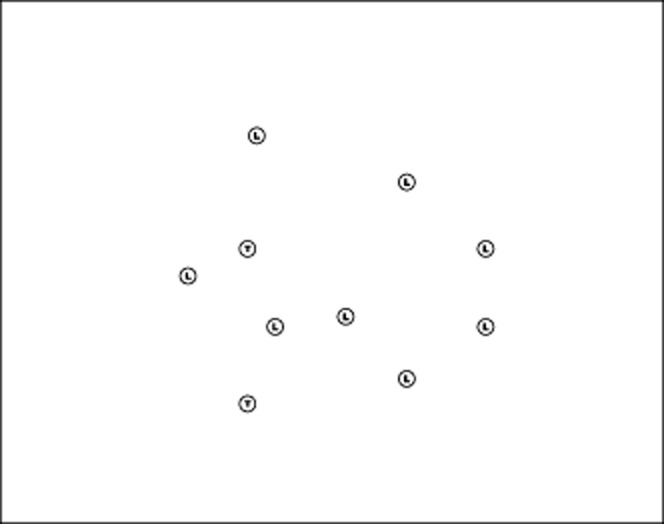
Example of a search display with two targets (T) and eight distractors (L). Stimuli were presented in white on a black background. Figure is not true to scale; exact dimensions are given in the Method section.

Stimuli were presented in white on a black background. We used “T” for target letters and “L” for distractors. There were always ten letters in the display; either one or two of them were targets, the remaining letters were distractors. The letters subtended an area of 0.32° × 0.32° at a viewing distance of 63 cm. The items appeared randomly at the intersections of an imaginary 6 × 6 grid. The size of a grid cell was 3.6°. The center of the letter deviated randomly from the intersection by ± 0.23° both in horizontal and vertical direction. The whole viewing area subtended 21.6° × 21.6° (see Figure 1). This display layout ensured that adjacent items were separated by at least 3°. Letters were surrounded by white circles. The outer diameter of the circle was 0.9° with a line thickness of 0.18°. The circle around the letter served two purposes. It reduced the ability to identify the letter without fixation (Bouma, 1970) and provided a clear target for the planning of the saccade. In a pilot experiment, we demonstrated that letter identification did not differ reliably from chance when fixation was more than 3° away from these stimuli (Körner & Gilchrist, 2007).

Participants were seated in a dimly lit, acoustically and electrically shielded booth in front of the monitor with a viewing distance of 63 cm. Before the start of the experiment, participants practiced on ten sample displays. The experiment consisted of a session of three blocks of 120 trials each with short breaks between blocks. Trial order was randomized across participants with an equal number of one- and two-target displays within each block. At the beginning of each trial, a fixation point was presented in the center of the screen. The trial was started only when fixation on the fixation point was registered. Participants were instructed to decide whether there were either one or two targets in the display. The search display was presented until the participant pressed one of two buttons on a response box. Participants were told to use the right hand for a two-target response and the left hand for a one-target response. Participants were instructed to respond as quickly and as accurately as possible.

### Apparatus

#### Eye movement recordings

We recorded two-dimensional eye movements using an Eye-Link I eye tracker (SR Research, Canada; SensoMotoric Instruments, Germany). The eye tracker is a head-mounted system that uses two infrared cameras that monitor the eyes at a sampling rate of 250 Hz. It also uses a head**-**movement compensation mechanism. To reduce pressure on the EEG electrodes, we padded the EEG cap with foam material before setting up the eye tracker on top of it. We calibrated both eyes and recorded from the eye that produced the better spatial resolution, which was typically better than 0.35°. Displays were presented on a 17-inch monitor with a resolution of 1,280 × 1,024 pixels. A chin rest was used to minimize head movement. The velocity threshold for saccade detection was set to 35°/second, the acceleration threshold was set to 9,500°/second^2^. Fixations were defined by the absence of a saccade. The eye tracker was calibrated before each of the three blocks using a 9-point calibration procedure. A drift correction (operated by the experimenter) was performed before each trial.

#### EEG recordings

Scalp potentials were recorded from 24 EEG positions according to the extended 10–20 system using Ag/AgCl electrodes. All electrodes were referenced to the left mastoid. For EEG recordings, two g.USBamp amplifiers (g.tec, Austria) were used. Horizontal electrooculogram (EOG) was recorded from electrodes placed on the outer canthi, and vertical EOG was recorded by electrodes placed above and below the right eye. EEG and EOG signals were digitized with a sampling rate of 512 Hz and prefiltered with a band-pass filter ranging from 0.1 Hz to 70 Hz. Electrode impedances were kept below 5 kΩ for the EEG recording and below 10 kΩ for the EOG recording. To synchronize EEG recordings and eye tracking, a TTL trigger signaling the end of a trial was sent from the eye tracker to the EEG recorder via the g.TRIGbox trigger box (g.tec, Austria). On the basis of this synchronizing trigger and the eye tracking data, triggers for the individual fixations were calculated and coded (reflecting information about display type, participant response, and the fixation's sequential position relative to the first target fixation) offline and inserted into the EEG data file. This method ensured a synchronization of the EEG and the eye-tracking data.

### Data Preprocessing and Analysis

For each participant, we collected 180 trials for the one-target and 180 trials for the two-target search condition. In total, 24 trials (0.4%) were deleted because they were not completely recorded by the eye tracker due to technical malfunction. In total, participants responded incorrectly in 163 trials (2.7%, range: 0.6%–6.1% across participants). These trials were also deleted from all analyses.

**Figure 2:**
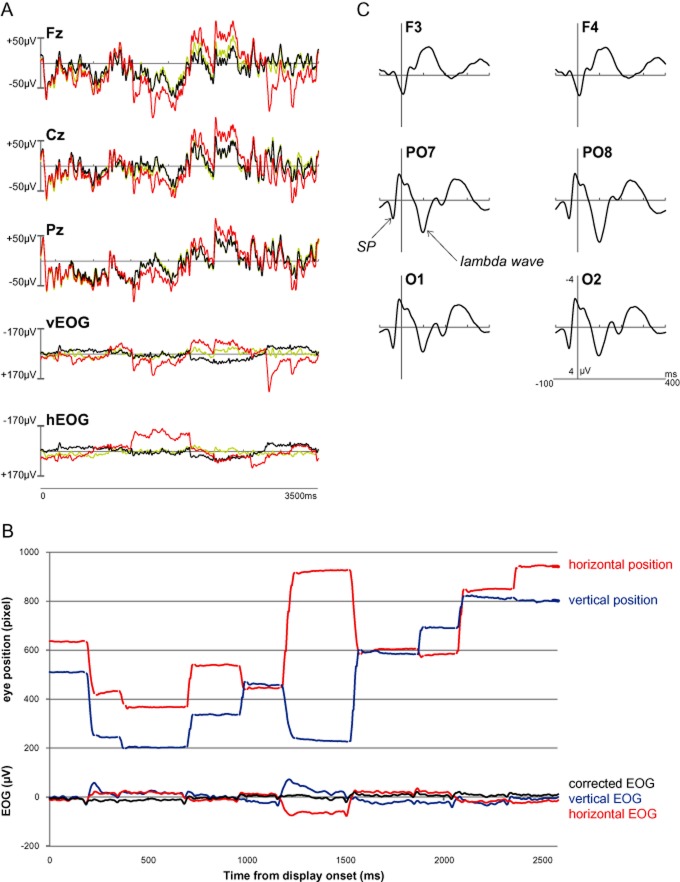
A: Example EEG (Fz, Cz, Pz) and EOG data (vertical EOG and horizontal EOG) from a representative trial before correction of ocular artifacts (in red), and after correction using Infomax ICA (Makeig et al., 1997; in green) and a regression-based method (Gratton, Coles, & Donchin, 1983; in black). B: Example eye tracking data (top) from a single trial synchronized with the corrected and uncorrected EOG (bottom). C: Main FRP components (spike potential, SP; lambda wave) illustrated for electrode sites for which they were most prominent.

EEG data preprocessing and analysis was performed with the Brain Vision Analyzer 2 software package (BrainProducts GmbH, Munich, Germany). We considered two correction methods for ocular artifacts (blinks and eye movements), an algorithm based on linear regression (eye movement correction procedure EMCP) without raw averaging (Gratton, Coles, & Donchin, 1983) and Infomax independent component analysis (ICA; Makeig, Jung, Bell, Ghahremani, & Sejnowski, 1997). The EMCP without raw averaging can remove about 80% of EOG artifacts (Schlögl et al., 2007). For both correction methods, the EOG channels on the outer canthi of both eyes and above the right eye were treated as bipolar channel pairs (“above-left,” “right-above”), thus separating horizontal and vertical eye movements. After correction, the EOG channel and its reference channel have the same signal. We found that the differences in the corrected EEG signal from the two methods were minimal (see Figure 2A) and thus used EMCP because it did not require user interaction. A sample eye track with EOG data before and after EMCP is shown in Figure 2B.

At this stage, EEG artifacts were automatically marked for later deletion (criteria: voltage values exceeding ± 70.00 μV, a change of more than ± 50.00 μV per sampling step, no voltage values below ± 0.50 μV over an interval of 100 ms). Afterwards, the EEG data were filtered (0.1 Hz to 30 Hz band-pass phase shift-free Butterworth filter, slope: 24 dB/oct, 50 Hz notch filter).

Each trial could contain a maximum of five fixation events of interest: first fixation on the first target (T), fixation on the last distractor before fixating on the first target (−1D), fixation on the first distractor after T (+1D), fixation on the second distractor after T (+2D), fixation on the third distractor after T (+3D). For each of these fixation event types, we computed average FRPs per electrode position and participant for an epoch starting 100 ms before fixation (onset of fixation as determined by the eye tracker). An epoch was excluded from analysis if it contained artifacts as defined above. This resulted in the loss of 380 epochs in total (6.2%).

In order to minimize the distorting influence of eye movements on the FRPs, we limited the analyzed epochs for comparison of distractor fixation events to a period during which the eyes were not moving, that is, the period when the eyes were fixating. Specifically, we analyzed FRPs within 200 ms after the onset of a distractor fixation (fixation-related potentials) and 200 ms before the offset of that fixation, that is, the onset of the following saccade (saccade-related potentials, SRPs). The shortest mean fixation duration of any distractor fixation event in this experiment was 208 ms (see Table 1), that is, it was longer than an analyzed epoch. Therefore, the analyzed FRPs/SRPs are virtually unaffected by components that originate from eye movements themselves and also from eye movement-related components such as the presaccadic spike potential. Thus, the combination of FRP and SRP for a given fixation event provides a complete description of the brain's activity during fixation. Furthermore, there is almost no temporal overlap between the FRPs of successive fixation events (+1D, +2D, +3D).

**Table 1:** Fixation Duration, Amplitude of Preceding Saccade, and Average Number of Analyzed Events for Each Fixation Event Type

Fixation event type	Mean fixation duration (*SD*) in ms	Mean saccade amplitude (*SD*) in degree v.a.	Average number of analyzed events (*SD*) per participant
−1D	214 (36) / 214 (32)	5.71 (0.70)	129.12 (26.44)
T	250 (47) / 240 (42)	6.40 (0.41)	123.88 (21.31)
+1D	230 (24) / 229 (27)	7.50 (0.80)	116.82 (17.69)
+2D	210 (28) / 208 (29)	5.89 (0.78)	108.24 (16.43)
+3D	208 (26) / 208 (27)	6.08 (0.56)	99.82 (14.50)

*Note.* Mean fixation durations are given based on the individual means (before slash) and on the individual medians (after slash). v.a. = visual angle.

Fixation-related potentials entered the analysis only if the following conditions were met: (a) To avoid a contamination by the onset of the display, only events after the first three fixations in a trial were considered; (b) The manual response did not occur within 600 ms after the beginning of an event; (c) Within 600 ms after the beginning of the −1D and T events, there were no refixations of these items, and within a 600 ms period after beginning of the +1D, +2D, and +3D events, there was no target (re-)fixation. Due to these precautionary measures, from a theoretical maximum of 360 epochs per event and participant, an average of 115.6 epochs per event and per participant (32.1%) remained for ERP analysis (see Table 1 for the average number of epochs per event).

To avoid a loss of statistical power, only the three midline electrode positions (Fz, Cz, Pz) were analyzed using repeated measures analyses of variance (ANOVAs). The Greenhouse–Geisser correction (G-G corrected *p* values are reported) was always applied when reporting effects in repeated measures ANOVAs with more than one degree of freedom in the numerator (Geisser & Greenhouse, 1959).

The choice of an appropriate baseline in FRP analysis is challenging. The FRPs reported in the Results section were calculated on the basis of a common baseline using the interval of -100 ms to 0 ms before the T event for all fixation event types (−1D, T, +1D, +2D, +3D). This baseline has the advantage of being temporally close to the analyzed events. Importantly, it provides a common reference point for the comparison of distractor fixations before (−1D) and after target fixation (+1D, +2D, +3D). However, this baseline overlaps partially with the tail of −1D potential. One alternative analysis would therefore involve a baseline period from the beginning or the end of a trial. However, this kind of baseline has the disadvantage of being temporally distant to the analyzed events. We have included analyses with such a baseline in the Supporting Information. Another alternative analysis involves baselines that are calculated separately for each event type. However, in the present case this can lead to a bias in the estimation of the duration and amplitude of an ERP. For example, with regard to the expected sustained negative shift in brain activity after target fixation (event types +1D, +2D, +3D), a separate baseline would reset the level of all FRPs to zero. Thus, a sustained negativity is underestimated, if it is artificially reset to zero in the baseline calculation for the next fixation. Similarly, the negativity for the +1D fixation is overestimated because of the expected positivity of the preceding target fixation. As a control, we also included analyses using a separate baseline in the Supporting Information. These control analyses showed that our findings are robust with regard to the choice of baselines.

## Results

### Manual Response Times and Eye Movements

We computed manual response times and number of fixations for all correct trials. The average correct manual response time (averaged across individual means) in the one-target search was 3,571 ms (*SD* = 486 ms) and 2,771 ms (*SD* = 374 ms) in the two-target search. The corresponding average number of fixations recorded per trial was 14.74 (*SD* = 1.79) and 11.19 (*SD* = 1.20), respectively. Both differences were statistically significant, *t*(16) = 18.36, *p* < .001, 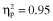, and *t*(16) = 15.65, *p* < .001, 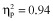, respectively. A high covariation between manual response times and number of fixations is typical in serial visual search (Williams, Reingold, Moscovitch, & Behrmann, 1997). Participants needed more time and made more eye movements in the one-target search because they had to inspect all of the items in the display before they could respond. In the two-target search, they could abort the search and respond as soon as they had found the second target.

To identify the item fixated, we calculated, for each fixation, the Euclidean distance between that fixation and each item in the display of a trial. The fixation was then attributed to the item with the smallest distance. This is an established method in eye movement research (see, e.g., Zelinsky, 1996). As an alternative, we assigned a fixation to an item only if the fixation was within a 2° box surrounding the center of that item. This did not change the original assignment; however, 9% of fixations could not be assigned according to this criterion. We therefore preferred the least-distance method, to avoid further data loss. This classification left in total 57 trials (1.88%) for which none of the fixations could be assigned to the target in the one-target condition and in total 55 trials (1.89%) for which none or only one of the fixations could be assigned to the targets in the two-target condition. These trials were removed from the following analyses. On average, participants fixated the first target after 6.78 fixations (*SD* = 0.63) in the one-target search and after 4.60 fixations (*SD* = 0.44) in the two-target search, *t*(16) = 21.54, *p* < .001, 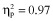. The expected number of successive search steps for finding a target in a 10-item display is 5.5 if there is only one target present; the corresponding number for two targets is 3.67 (e.g., Hogg & Craig, 1989), resulting in a ratio of 1.5. The observed ratio of 1.47 was close to this theoretical ratio suggesting that participants scanned both types of such displays in a serial manner. Figure 2B shows an exemplary eye track for a one-target trial, synchronized to the uncorrected and the corrected EOG. The figure illustrates the close concordance of eye position changes and corresponding changes in the potential difference of the EOG.

We computed fixation durations and saccade amplitudes for those fixation events that were later used in the analysis of FRPs (−1D, T, +1D, +2D, +3D). The results are presented in Table 1. Fixation durations differed between event types, *F*(4,64) = 25.92, *p* < .001, 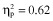. Specifically, T fixations lasted longer than any of the distractor fixations, and the +1D fixation lasted longer than any of the remaining distractor fixations (*p*s < .01, Newman-Keuls post hoc tests). The amplitudes of the saccades that preceded the respective fixations also differed, *F*(4,64) = 37.20, *p* < .01, 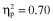. The saccade amplitude preceding fixation +1D was greater than any other amplitudes, and the saccade preceding the T fixation was greater than the saccades preceding the −1D and +2D fixations (*p*s < .01, Newman-Keuls post hoc tests).

### Fixation-Related Potentials

To illustrate two characteristic components, Figure 2C shows selected FRPs for lateral frontal, parieto-occipital, and occipital electrode positions, averaged across all fixation event types: (a) For lateral parietal and occipital electrode positions, there was a potential change at around 30 ms prior to fixation. This is the presaccadic spike potential that coincides with saccade onset and reflects electrical activity, which may be caused by the firing of oculomotor neurons or by the muscles (Boylan & Ross Doig, 1989; Riemslag, van der Heijde, van Dongen, & Ottenhoff, 1988); (b) After fixation onset, there was a distinct positive-going wave, which peaked at approximately 100 ms. This is the so-called lambda wave (Kazai & Yagi, 2003), which is considered a visually evoked potential that represents information inflow at fixation onset (Thickbroom, Knezevich, Carroll, & Mastaglia, 1991). As typically observed, this component was present for lateral parietal and occipital electrode sites but not for frontal sites.

**Figure 3:**
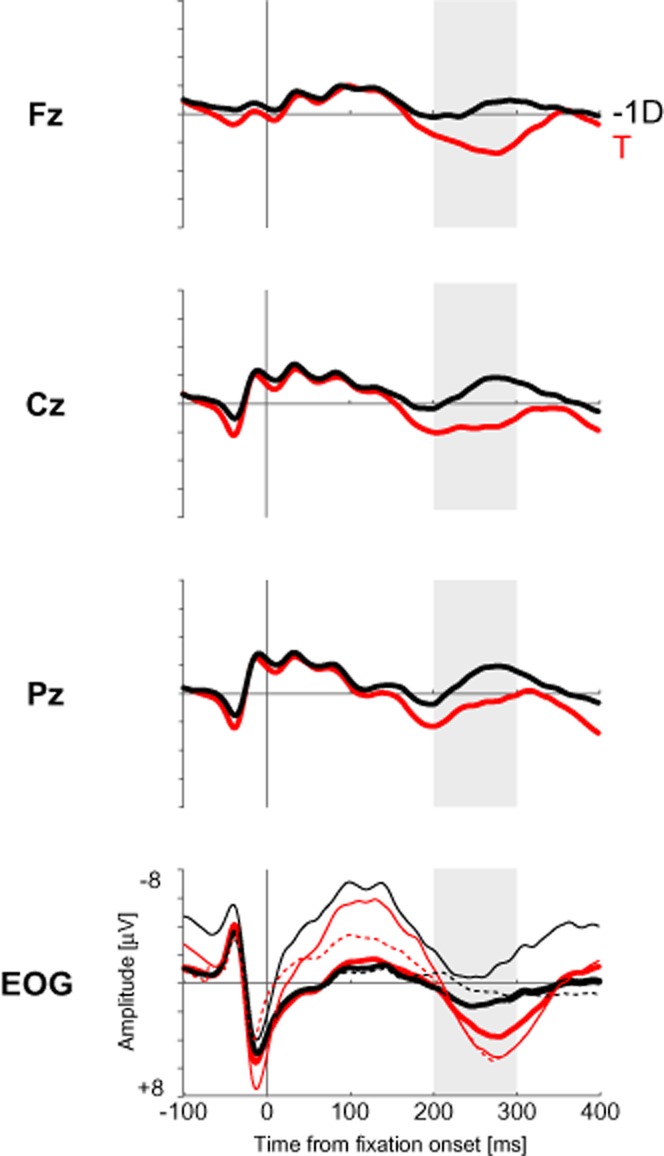
Fixation-related potentials for fixations on the first target T (in red) and fixations on the last distractor −1D (in black) before fixating the first target, for midline electrodes. Bipolar EOG channels for T (in red) and −1D (in black) before (vEOG, dashed lines and hEOG, thin solid lines) and after EOG correction (thick solid lines).

The FRPs for T and −1D fixations are shown in Figure 3. FRPs differed between fixations on the first target (T) and fixations on the last distractor before target fixation (−1D). The FRP on target fixations showed a positive-going wave that was maximal over frontocentral electrodes in a time window between 200 and 300 ms. Because a P3 could be expected on targets as relatively rare events, we illustrate this positivity in Figure 3. It should be noted, however, that the T fixation event produced a positive deflection in the corrected EOG also. This indicates that the positivity observed is possibly confounded with residual eye movements. We are, therefore, reluctant to interpret this positivity as a P3.

**Figure 4:**
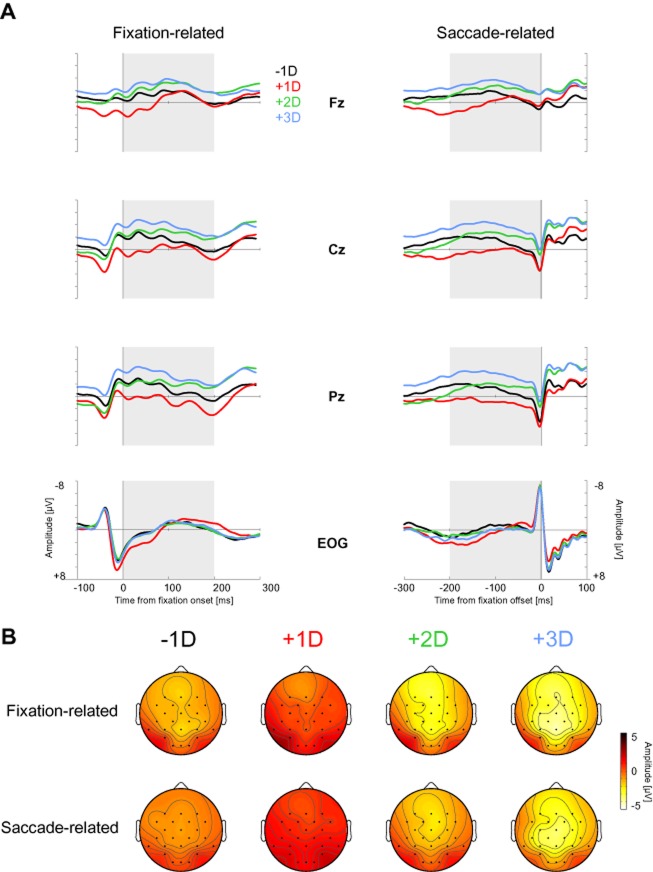
A: Fixation-related potentials (FRPs) for distractor fixations before (−1D, in black) and after the first target fixation (+1D in red, +2D in green, +3D in blue), aligned to the onset (FRP, left column) and offset (saccade-related potentials [SRPs], right column) of the respective fixation for midline electrodes and corrected vertical and horizontal EOG. Gray rectangles denote the 200-ms time windows for the negativity analysis. B: Scalp topographies for the FRPs (top row) and the SRPs (bottom row) for the distractor fixations before and after target fixation, during the time windows from Figure 4A.

Fixation-related potentials of distractors fixated before and after the first target fixation also differed from each other, indicating that the detection of the target influenced the processing of subsequent distractors in continued visual search. We analyzed FRPs and SRPs for distractor fixations within a 200-ms time window (see Data Preprocessing and Analysis section). FRPs and SRPs and their topographies for the distractor fixation events are shown in Figure 4A and 4B, respectively.

With respect to FRPs (Figure 4A, left), the fixations for the second and third distractor after the target fixation (+2D and +3D) produced a consistently more negative potential than the last distractor fixation before the target fixation (−1D) for all electrode positions and throughout the analyzed interval. The first distractor fixation after the target fixation (+1D) showed a potential that started out more positively than the −1D potential but approached it for electrode positions Fz and Cz during the analyzed interval.

To evaluate these differences statistically, the mean values of the amplitudes within the 200-ms window after fixation onset were entered into a repeated measures ANOVA with the within-subject factors fixation event type (−1D, +1D, +2D, +3D) and position (Fz, Cz, Pz). Compared with the −1D average amplitude, the +1D amplitude was more positive (in particular for position Pz) while the two subsequent amplitudes (+2D and +3D) were more negative. As expected, this resulted in a significant main effect fixation event type, *F*(3,48) = 45.70, *p* < .001, 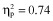 and an interaction Fixation Event Type × Position, *F*(6,96) = 7.23, *p* < .001, 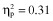. No further effects were significant. The fixations of +2D and +3D elicited a larger negativity than the −1D fixation and the +3D amplitude was more negative than the +2D amplitude, while the +1D amplitude was more positive than all the other amplitudes (*p*s < .01, Newman-Keuls post hoc tests).

With respect to SRPs, an identical pattern of results emerged (Figure 4A, right). During the last 200 ms of the distractor fixations, the +2D and +3D potentials were more negative than the −1D potential, while the +1D potential was more positive (particularly for electrode position Pz). This produced the same statistical pattern as the FRPs: a significant main effect fixation event type, *F*(3,48) = 46.55, *p* < .001, 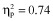, and an interaction Fixation Event Type × Position, *F*(6,96) = 5.63, *p* < .001, 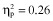. The fixations of +2D and +3D elicited a larger negativity than the −1D fixation and the +3D amplitude was more negative than the +2D amplitude, while the +1D amplitude was more positive than all the other amplitudes (*p*s < .01, Newman-Keuls post hoc tests).

The positivity of the +1D fixation might be a carryover effect of the above reported positivity for target fixations that precede the +1D fixation (see Figure 3). Visual inspection of the +1D potential in Figure 4A (left) suggests that it has approached the −1D potential after 100 ms, at least for frontocentral electrode sites. To test this, we carried out an ANOVA of the above type, restricted to the latter half of the time window (100–200 ms). All three effects were significant: main effect fixation event type, *F*(3,48) = 29.34; *p* < .001, 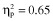, main effect position, *F*(2,32) = 14.71; *p* < .001, 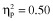, and interaction Fixation Event Type × Position, *F*(6,96) = 15.66, *p* < .001, 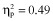. Newman-Keuls post hoc tests revealed that the difference between the +1D and the −1D mean amplitude was not significant for position Fz (*p* = .75) but was significant for positions Cz and Pz (*p*s < .001). In sum, these results indicate that having fixated the target influenced subsequent distractor processing at least for as long as three consecutive fixations.

We compared FRPs for distractor events before and after target fixation using two additional types of baselines: (1) a baseline period before display onset, and (2) separate baselines. The basic pattern of a posttarget distractor negativity did not change (see Supporting Information). It must be noted that the average amplitude of the preceding saccade differed between the event types (see Table 1). We therefore reran these analyses and controlled for saccade amplitude (see Supporting Information). These additional analyses demonstrated that the effect of distractor negativity after target fixation is robust. It did not depend on the choice of a particular baseline, nor did it vary within the range of the observed saccade amplitudes.

## Discussion

In the present experiment, we investigated whether finding a target changed subsequent distractor processing in continued serial search. Participants were asked to decide whether a search display contained one or two targets, while being allowed to move their eyes freely from item to item. Eye movements were recorded and EEG potentials were averaged for target and distractor fixations (FRPs).

Consistent with previous results on continued serial search, participants needed more fixations and produced longer response times in one-target displays than in two-target displays, indicating that participants could stop the search once the second target was found, before having inspected all items of the display. FRPs on the first target were accompanied by a positive-going wave when compared to the FRP of the last distractor fixation before finding the first target. Furthermore, after having fixated the first target, subsequent distractor FRPs showed a negative shift when compared to the last distractor fixation before finding the first target. This difference in FRPs shows that having detected the first target changed the processing of subsequent distractors, possibly reflecting processes related to working memory.

A first finding was a positive-going wave in the FRP of the first target fixation compared to the fixation on the last distractor starting 200 ms after fixation onset. For earlier time points, the FRPs of the first target and the last distractor were basically identical. The positivity in the target FRP peaked between 200 and 300 ms and the peak differed with regard to topography, with a more frontocentral distribution. A P3 was expected for target fixations compared to distractor fixations (Luck & Hillyard, 1994; Sutton et al., 1965). The detection of a target is a task-relevant event that requires an update or a revision of the mental representations involved in the task. This is consistent with an interpretation of the P3 as an index of context updating (Donchin, 1981; Polich, 2007).

However, the corrected EOG showed a difference between T and −1D event types that is similar to the target positivity (see Figure 3). It seems therefore possible that residual eye movement components are responsible for it. We had expected a P3 on the basis of previous EEG research on visual search because fixating a target should be a rare event. In our experiment, participants fixated 6.94 and 4.70 distractors before finding the first target, in a one-target display and two-target display, respectively. It is possible that targets were not infrequent enough in our 10-item displays to give rise to a distinct P3.

Our second (and main) finding was that distractor processing was altered after having fixated the first target. For the second and third distractor fixation after the target (+2D, +3D), we observed a negative shift compared to the last distractor fixation before the target (−1D). The potential for the first distractor fixation after the target fixation started out more positively than the −1D potential but approached it for position Fz. These results suggest that having found the first target causes a longer negative shift in the EEG that remained at least for three distractor fixations following the target. The observed pattern is compatible with a gradual buildup of the negativity from the first to the third distractor fixation after having fixated the target. This could indicate that the mental process causing it needed time while the search process continued. We had expected a negative shift after fixating the first target, because the task requires participants to memorize the detection of the first target and its location, while continuing the search until either a second target was found or all items had been inspected. Previous EEG research using a delayed match-to-sample task observed a NSW during the retention period (Barret & Rugg, 1988, 1989; Ruchkin, Johnson, Canoune, & Ritter, 1990). The NSW was observed to increase with memory load (Mecklinger & Pfeifer, 1996; Ruchkin et al., 1997) and to correlate positively with retrieval probability (Rösler et al., 1997). In the present experiment, the search had to continue after finding the first target and the response could not be prepared before encountering the second target or after having visited all items in the display. To isolate memory-specific components of the NSW in delayed match-to-sample tasks, several ERP studies have exploited lateralization differences of the NSW when visual information is presented in the left or right visual field (contralateral delay activity [CDA]; Klaver et al., 1999).

Perez and Vogel (2012) state that the CDA as well as the NSW take approximately 450 ms to reach maximum. In the present study, we observed a gradual buildup of negativity across the first and second distractor fixation after the target, compared to the last distractor fixation before the target. There was also a negativity present for the third distractor after the target, indicating a sustained negativity. We would expect a sustained negativity, or NSW, for the entire period from the target fixation until the end of the trial. On the other hand, it is also possible that the processing of visual input from distractor fixations might interfere with retention processes during that interval, as the paradigm we used is different from match-to-sample paradigms, where no stimulus is presented in the retention interval. Although further studies are needed to assess the memory specificity of the negative shift observed here, it is highly likely that it reflects memory processes during continued serial visual search.

A third finding is the systematic differences in the eye tracking data between target and distractor fixations. Fixation of the target and the first distractor after the target (+1D) showed a longer fixation duration compared to all other distractors. The saccade amplitude after the target fixation was greater than any other saccade amplitude. The results show that processing the target influenced significantly the programming of subsequent saccades and fixations. To our knowledge, such differences have not been reported before and are remarkable in their own right.

Recently, there has been a controversy about the extent to which visual search relies on memory processes. Horowitz and Wolfe (1998, 2001) have argued that search is amnesic by nature. However, others have presented evidence from behavioral measures indicating that memory processes support visual search by tagging targets and distractors that have already been fixated to avoid unnecessary refixations (e.g., Gibson et al., 2000; Körner & Gilchrist, 2007). Our finding of lasting sequential effects on distractor processing after finding the first target indicates a change in brain activity that is most likely due to the memorization of the first target.

It is possible that the sequential effects we observed are not only due to memory processes alone. Every distractor fixation after fixating the first target increases the likelihood that a manual response must be initiated next. Finding the first target corresponds to the end of the first phase of the task and could lead to a higher level of response preparation for the remainder of the trial. It is therefore possible that the electrophysiological results observed here reflect not only retention but also processes signaling the end of the task. Our results so far, however, are consistent with the view that memory processes play a role in serial visual search.

In this experiment, we have applied the technique of fixation-related potentials to serial visual search for the first time. Our participants were allowed to move their eyes freely from item to item. By recording these eye movements, we were able to track the deployment of visual attention. Locking ERPs to fixations and saccades allowed us to follow the brain's response on a moment-by-moment basis so that we could compare target and distractor processing in different phases of the task. Our results indicate that fixation-related potentials provide a valuable tool to track the dynamic changes in brain activation and cognitive processing during a complex task such as continued serial visual search.

The measurement and analysis of FRPs present a number of challenges because the EEG signal is prone to distorting influences from eye movements. Typically, epochs of EEG are rejected when they are contaminated by eye movements. However, this is not an option when, as in our study, participants are required to move their eyes. We decided to use a regression approach to correct for eye movement artifacts (Gratton et al., 1983). This is an objective and conventional method, although it cannot eliminate all eye movement artifacts. ICA is an alternative (Jung et al., 2000) but has the disadvantage that often the identification of components related to eye movements is left to the experienced experimenter (see Mognon, Jovicich, Bruzzone, & Buiatti, 2011, for a recent exception). Multiple-source eye correction (Ille, Berg, & Scherg, 2002) can also be used for artifact correction (Dimigen et al., 2011) but requires the recording of calibration eye movements.

Another problem is the overlap between epochs that last longer than the duration of a fixation. One method to account for this overlap is the adjacent response (ADJAR) technique (Woldorff, 1993). Although this is a valid method, it can change the original EEG signal considerably. We have therefore opted to restrict, wherever possible, the analysis of FRPs to periods that were unlikely to overlap with adjacent saccades and fixations. We consider our careful and conservative approach to FRPs in serial search as appropriate.

We designed this experiment with the goal of finding an electrophysiological marker of item processing in continued serial visual search. To this end, we measured eye fixation-related potentials in visual search for the first time. We are aware that this experiment can only be the beginning of a longer-term endeavor with the goal to work out the functional specificity of the FRPs observed.

## Supporting Information

Appendix S1 This section provides additional analyses using different baselines and controlled saccade amplitude.

Figure S1 Fixation-related potentials (FRPs) for distractor fixations before and after the first target fixation. A subset of data with comparable saccade amplitudes and a baseline period before display onset was used.
