# Supplementary material for: Sequential effects in continued visual search: Using fixation-related potentials to compare distractor processing before and after target detection
Source: Psychophysiology. 2014 Feb 11;51(4):385–95. doi: 10.1111/psyp.12062 (PMC4283708; doi:10.1111/psyp.12062)
Supplement: Appendix S1 — This section provides additional analyses using different baselines and controlled saccade amplitude. [file psyp0051-0385-SD1.docx]

**Supporting Information**

The choice of an appropriate baseline in FRP analysis is challenging. In the Results section, we reported a negativity for distractor fixations that occurred after the target fixation. The respective FRPs were calculated on the basis of a common baseline (see Figure 4A, left). To show that the distractor negativity is present with different choices of baselines we present the following analyses.

First, we used a 100-ms blank period after the registration of the fixation and before display onset at the beginning of a trial (see Stimuli and Procedure section) as common baseline. This baseline does not overlap with any of the analyzed epochs. The resulting potentials looked very similar to those depicted in Figure 4A (left). The only difference with respect to the original analysis was a general negative shift for electrode positions Cz and Pz. (This shift is illustrated in Figure SI1, left, for a subset of data with equal saccade amplitude; see below.) Accordingly, a repeated measures ANOVA with factors fixation event type (–1D, +1D, +2D, +3D) and position (Fz, Cz, Pz) resulted in a significant main effect fixation event type, *F*(3, 48) = 45.74, *p* < .001, η_p_^2^ = 0.74, and a main effect of position, *F*(2, 32) = 6.45, *p* < .05, η_p_^2^ = 0.29, as well as an interaction Fixation Event Type × Position, *F*(6, 96) = 7.23, *p* < .01, η_p_^2^ = 0.31. The FRP for the fixation of distractor +1D was more positive than for the other distractor fixations. Importantly, potentials for fixation events +2D and +3D elicited a larger negativity than the other distractor fixations and the potential for fixation event +3D was more negative than the +2D fixation potential (*p*s < .01, Newman-Keuls post hoc tests). The general negative shift at electrode positions Cz and Pz is probably caused by the fact that this baseline period is temporally more distant from the analyzed epochs than the period that was used in the analyses reported in the results section.

In a second alternative analysis, we used the period of -100 to 0 ms before each fixation event type as separate baselines. The fixations for the first, second and third distractor after the target fixation (+1D +2D, and +3D) produced a more negative potential than the last distractor fixation before the target fixation (-1D), in particular for positions Fz and Cz. The FRP of the third distractor fixation (+3D) was more negative than that of +1D and more positive than +2D; this was more pronounced for position Pz than for positions Fz and Cz. To evaluate these differences statistically, the mean values of the amplitudes within the 200-ms window after fixation onset were entered into a repeated measures ANOVA with factors fixation event type (–1D, +1D, +2D, +3D) and position (Fz, Cz, Pz). This resulted in a significant main effect of fixation event type, *F*(3, 48) = 10.64, *p* < .001, η_p_^2^ = 0.40, and position, *F*(2, 32) = 4.23, *p* < .05, η_p_^2^ = 0.21, and an interaction Fixation Event Type × Position, *F*(6, 96) = 15.72, *p* < .001, η_p_^2^ = 0.50. The fixation of +2D elicited a statistically larger negativity than all the other distractor fixations (*p*s < .01, Newman-Keuls post hoc tests). In sum, these analyses show that the reported effect of distractor negativity is robust and does not depend on the choice of a particular baseline.

Saccade amplitudes differed between fixation event types (Table 1). Saccade amplitude is related to the pre-saccadic spike potential and the lambda wave (Boylan & Ross Doig, 1989; Yagi, 1979), and Graupner et al. (2011) have recently reported effects of saccade amplitude on other FRP components. It is also known that saccade amplitude decreases in long visual search trials (Over, Hooge, Vlaskamp & Erkelens, 2007). It is therefore possible that varying saccade amplitude systematically contributed to the differences reported for distractor fixations before and after target fixation. To test for the influence of such confounds we removed epochs with long saccades (amplitudes greater than 8° v.a.) and short (corrective) saccades (amplitudes smaller than 2° v.a.) from the original data set. The reduced data set contained 60.2% of the epochs of the original analysis and the distractor event types differed only marginally with respect to saccade amplitude, *F*(3, 48) = 2.47, *p* = .08 (Greenhouse-Geisser corrected). For this data set, we carried out the FRP/SRP comparison for distractor potentials before (-1D) and after target fixation (+1D, +2D, +3D). (To illustrate the negative shift caused by a baseline period which is temporally distant from the analyzed events we used the period of 100 ms after fixation registration and before display onset as a baseline; see above.) With respect to both FRPs and SRPs a pattern emerged that was almost identical with the pattern obtained for the original data set. In particular, the negativity for distractor fixations after the target fixation (+2D, +3D) compared with those before the target fixation (-1D) was present (see Figure SI1, left). Accordingly, a repeated measures ANOVA with factors fixation event type (–1D, +1D, +2D, +3D) and position (Fz, Cz, Pz) resulted in a significant main effect fixation event type, *F*(3, 48) = 31.97, *p* < .001, η_p_^2^ = 0.67, a main effect of position, *F*(2, 32) = 7.61, *p* < .01, η_p_^2^ = 0.32, and an interaction Fixation Event Type × Position, *F*(6, 96) = 4.44, *p* < .01, η_p_^2^ = 0.22. The potential for the event type +1D was more positive than for the other distractor fixations. Potentials for fixations of +2D and +3D did not differ from each other but importantly elicited a larger negativity than the other distractor fixations (*p*s < .01, Newman-Keuls post hoc tests).

With respect to SRPs, an almost identical pattern of results emerged (Figure SI1, right). During the last 200 ms of the distractor fixations the +2D and +3D potentials were more negative than the –1D potential. This produced the same statistical pattern as the FRPs: a significant main effect fixation event type, *F*(3, 48) = 35.50, *p* < .001, η_p_^2^ = 0.69, a main effect of position, *F*(2, 32) = 5.80, *p* < .05, η_p_^2^ = 0.27, and an interaction Fixation Event Type × Position, *F*(6, 96) = 6.39, *p* < .001, η_p_^2^ = 0.29. The SRP for the event type +1D was more positive than for the other distractor fixations. Importantly, potentials for fixations of +2D and +3D elicited a larger negativity than the other distractor fixations and the SRP for fixation +3D was more negative than the +2D potential (*p*s < .01, Newman-Keuls post hoc tests).

To investigate the potential influence of saccade amplitude more closely we subdivided the data set in categories of short saccades (amplitudes between 2° and 5° v.a.) and long saccades (amplitudes between 5° and 8° v.a.). Next, we carried out a repeated measures ANOVA with factors fixation event type, position and saccade amplitude (short, long) on the FRPs. Replicating the results of the previous analyses the main effect fixation event type was significant, *F*(3, 48) = 32.29, *p* < .001, η_p_^2^ = 0.67, as well as the main effect position, *F*(2, 32) = 7.27, *p* < .01, η_p_^2^ = 0.31, and the interaction Fixation Event Type × Position, *F*(6, 96) = 4.46, *p* < .01, η_p_^2^ = 0.22. Importantly, the main effect saccade amplitude was not significant, *F*(1, 16) = 0.24, *p* = .63. Only the interaction Position × Saccade Amplitude was significant, *F*(2, 32) = 4.97, *p* < .05, η_p_^2^ = 0.24, due to slightly more positive FRPs for long saccades at position Pz. The interaction Fixation Event Type × Saccade Amplitude was not significant, *F*(3, 48) = 1.31, *p* = .28, nor was Fixation Event Type × Position × Saccade Amplitude, *F*(6, 96) = 1.75, *p* = .17. The same analysis for the SRPs also confirmed the previous result. There were main effects for fixation event type and position, as well as an interaction Fixation Event Type × Position. None of the effects involving the factor saccade amplitude were significant.

The absence of an effect of saccade amplitude may be somewhat surprising. Graupner et al. (2011) had subdivided their FRP data using a similar criterion (saccades smaller than 4° vs. saccades greater than 4°) and found a respective effect of saccade amplitude. This effect, however, was observed for relatively early components, such as the N1 and P2. It seems that in the case of our data saccade amplitude does not systematically influence the relatively long-lived general negativity for distractor FRPs following the target fixation. To summarize, a negativity for distractor fixations subsequent to the target fixation exists compared to fixations that precede the target fixation even when saccade amplitude is controlled for.
