# Supplementary material for: Sequential effects in continued visual search: Using fixation-related potentials to compare distractor processing before and after target detection
Source: Psychophysiology. 2014 Feb 11;51(4):385–95. doi: 10.1111/psyp.12062 (PMC4283708; doi:10.1111/psyp.12062)
Supplement: Figure S1 — Fixation-related potentials (FRPs) for distractor fixations before and after the first target fixation. A subset of data with comparable saccade amplitudes and a baseline period before display onset was used. [file psyp0051-0385-SD2.pdf]

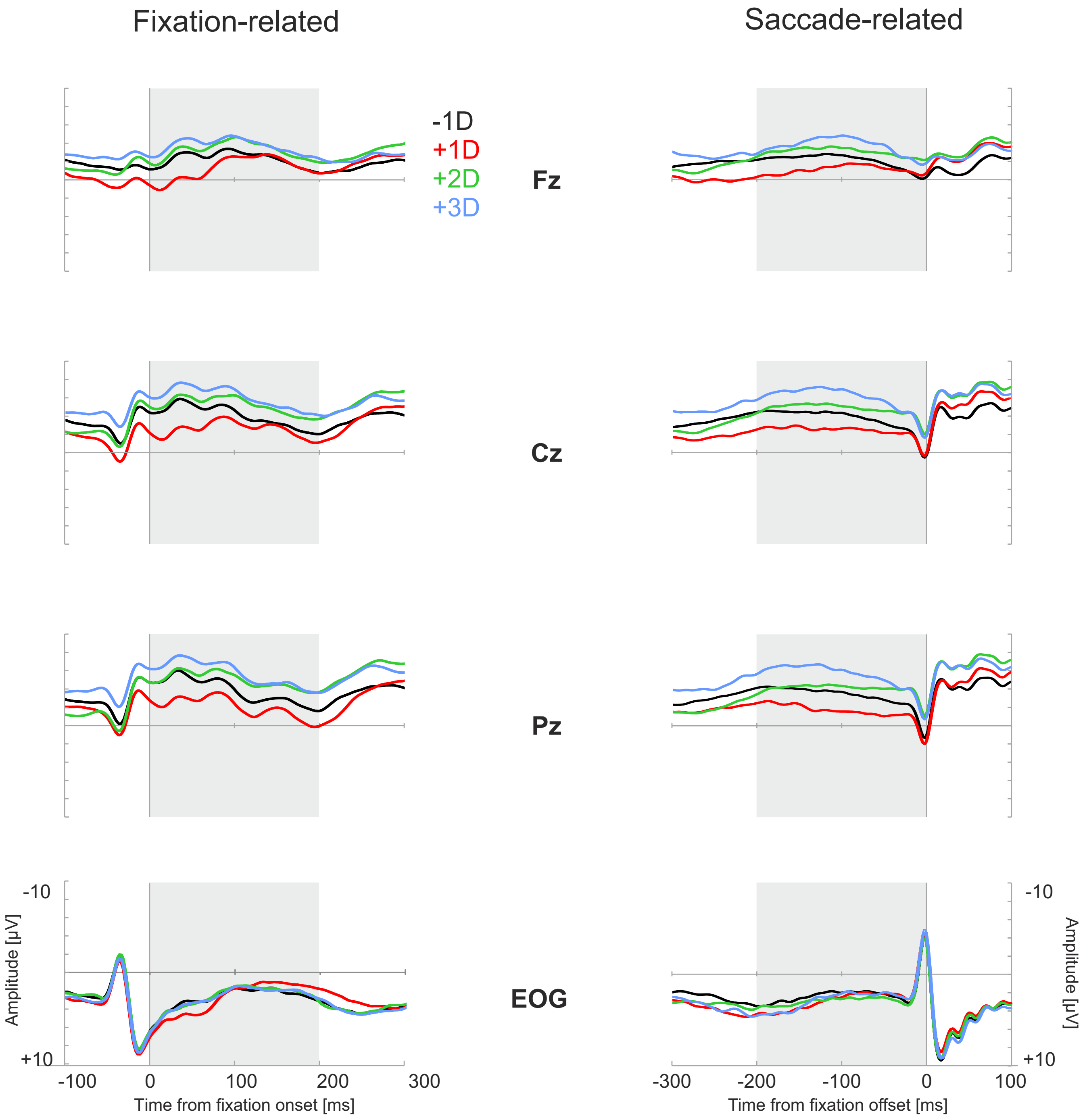

Figure SI1. Fixation-related potentials (FRPs) for distractor fixations before (–1D, in black) and after the first target fixation (+1D in red, +2D in green, +3D in blue), aligned to the onset (FRP, left column) and offset (saccade-related potentials [SRPs], right column) of the respective fixation for midline electrodes and corrected vertical and horizontal EOG. Grey rectangles denote the 200 ms time windows for the negativity analysis. A subset of data with comparable saccade amplitudes and a baseline period before display onset was used.

FIGURE SI1
